# Risk factors for antenatal hypovitaminosis D in an urban district in Malaysia

**DOI:** 10.1186/s12884-016-0939-3

**Published:** 2016-07-13

**Authors:** Noriklil Bukhary Ismail Bukhary, Zaleha Md Isa, Khadijah Shamsuddin, Khor Geok Lin, Zaleha Abdullah Mahdy, Haslinda Hassan, Noor Sharifatul Hana Yeop

**Affiliations:** Department of Community Health, Faculty of Medicine, Universiti Kebangsaan Malaysia Medical Centre, Jalan Yaacob Latif, Bandar Tun Razak, 56000 Cheras, Kuala Lumpur Malaysia; Petaling District Health Office, Wisma SAHOCA, No 1, Jalan SS 6/3A Kelana Jaya, 47301 Petaling Jaya, Selangor Malaysia; School of Health Sciences, Faculty of Medicine and Health, International Medical University, Bukit Jalil, 57000 Kuala Lumpur Malaysia; Faculty of Medicine, Department of Obstetrics and Gynecology, Universiti Kebangsaan Malaysia Medical Centre, Jalan Yaacob Latif, Bandar Tun Razak, 56000 Cheras, Kuala Lumpur Malaysia

**Keywords:** Prevalence, Pregnant women, Hypovitaminosis D, Risk factors, Ethnic, Skin type, Malaysia

## Abstract

**Background:**

Pregnant women form one of the high risk groups facing hypovitaminosis D. Low level of vitamin D will affect directly or indirectly both mother and fetus. Screening vitamin D in the first trimester of pregnancy is important to determine the necessary preventive action. Therefore, this study was aimed to determine the prevalence of hypovitaminosis D and its risk factors among pregnant women in the first trimester.

**Methods:**

A cross sectional study was carried out among first trimester pregnant women during their first antenatal visit. Samples were taken from different ethnicities in an urban district in Malaysia. A total of 396 respondents (99 % response rate) aged 18–40 years completed self–administered and guided questionnaire (characteristics and risk factors), validated semi-quantitative food frequency questionnaire for vitamin D in Malaysia (FFQ vitamin D/My), anthropometric measures (weight and height), blood test for serum 25(OH)D, skin measurement using Mexameter (MX 18) and Fitzpatrick Skin Type Chart Measurement (FSTCM). Data were analyzed to determine the association between risk factors and hypovitaminosis D.

**Results:**

The prevalence of hypovitaminosis D (serum 25(OH)D < 50 nmol/L) was 90.4 % (358). The mean age of respondents was 28.06 ± 4.09 years old. The independent predictors of hypovitaminosis D were Malay ethnicity (OR 33.68; 95 % CI: 12.81, 88.56), Indian ethnicity (OR 16.86; 95 % CI: 3.78,75.20), secondary education (OR 12.12; 95 % CI: 2.71, 54.16) and tertiary education (OR 14.38; 95 % Cl: 3.31, 62.45).

**Conclusion:**

Awareness should be raised among Malay and Indian pregnant women with secondary and tertiary education who consumed vitamin D (especially milk) poorly in order to prevent adverse health outcomes. Further studies need to be conducted among health care workers to determine their level of knowledge related to vitamin D, as they are front liner in detecting the hypovitaminosis D.

## Background

Malaysia is rich with sunshine, a major source of vitamin D. However, a study by Moy [1] among a cohort of Malays showed that 67.9 % had insufficient vitamin D especially in women, with serum mean concentration of 25(OH)D of 36.2 ± 13.4 nmol/L in women and 56.2 ± 18.9 nmol/L in men. Green et al. [2] reported that more than 60 % of women in Kuala Lumpur and Jakarta have serum 25(OH)D levels below 50 nmol/L. A study conducted by Khor et al. [3] among primary school children in Malaysia had shown lack of vitamin D with 70.4 % having serum 25 (OH) D <50 nmol/L. Lack of vitamin D may have an adverse health effects including death, cancer, cardiovascular disease, impaired immune function and glucose metabolism [4]. Vitamin D deficiency during pregnancy has been associated with problems such as maternal diabetes during pregnancy (GDM) [5–7] preeclampsia [8] and increased caesarean section [9].

Hien et al. [10] reported that vitamin D status among pregnant and non-pregnant women (reproductive age) living in Vietnam showed that the mean serum 25(OH)D differed between those living in urban and rural areas (78 nmol/L versus 85 nmol/L) with *p* = 0.02. Attention also must be given to those who have dark skin, less skin exposed to sunlight and obese because they have a higher risk of vitamin D deficiency warranting vitamin D supplement, mainly during pregnancy and lactation [11]. Meanwhile, Datta et al. [12] revealed that ethnic minorities (Asian) should be recommended for biochemical screening in early pregnancy, followed by supplement if low levels of vitamin D was found.

Vitamin D is an essential component of bone formation. It ensures sufficient absorption of calcium to build strong and healthy bones. Three important sources of vitamin D are sunlight, vitamin D supplements and food. Even though Malaysia is rich with sunlight, most Malaysians avoid exposing themselves directly to sunlight. Moreover, foods containing vitamin D is limited. Healthcare workers usually encourage pregnant women to increase calcium intake but they are less aware that without sufficient vitamin D, calcium intake will not have much effect especially during pregnancy. Therefore, it is necessary to determine the prevalence of hypovitaminosis D among pregnant women and assess its risk factors to overcome the problems caused by lack of vitamin D.

## Methods

### Subjects and sampling

A cross-sectional study was conducted in Petaling District, Selangor. This urban city is located in West Peninsular Malaysia, where the climate is consistently hot and humid throughout the year. This is different from East Coast Peninsular Malaysia where the rainy season occurs in the last three months of the year.

The subjects were recruited from three out of six government clinics in the Petaling District using purposive sampling. The selected clinics represented multiracial groups because of higher percentage of Chinese and Indian antenatal women compared to other government clinics in the Petaling District. It was then followed by proportionate stratified sampling based on earlier studies that have been carried out on a number of antenatal visits by pregnant women in the first trimester of each selected clinic. Sample size was calculated using Power and Sample Size Programme (PS2). The sample size required after considering the risk factors [13–15] and the outcome (hypovitaminosis D) [16] from previous studies were 381 participants. The figure was obtained after taking into consideration additional 20 % of possible non-response with 80 % power and α =0.05 (for 95 % CI). The inclusion criteria were Malaysian pregnant women in the first trimester of gestation aged between 18-40 years old. The exclusion criteria were mothers with pre-existing type I or type II diabetes, pre-existing hypertension, pre-existing parathyroid disease or uncontrolled thyroid disease, rheumatoid arthritis, metabolic bone disease, malabsorption, kidney disease, liver disease or treatment with antiepileptic drugs or antituberculous. Mothers with multiple pregnancies or who refused blood testing were also excluded from the study. These criteria were excluded as it would influence the vitamin D level.

The study was carried out from 1^st^ January until end of April 2014. A total of 818 eligible candidates were listed in the sampling frame. Next, the candidates were selected using odd numbers. Twenty two candidates refused to participate in the study. Meanwhile, 398 respondents were enrolled in this study. Respondents were given written informed consent and they were asked to complete the guided self-administered questionnaire on socio-demographic characteristics, risk factors and the validated semi-quantitative food frequency questionnaire (FFQ vitamin D/My). The validated FFQ contained food fortified with vitamin D, natural food rich with vitamin D and dietary supplement [17]. Meanwhile, Recommended Nutrient Intakes (RNI) for vitamin D among Malaysians was defined as ≥ 200 IU/day. They also underwent skin color measurement using the Fitzpatrick Skin Type Chart Measurement (FSTCM) and Mexameter (Mx) 18; weight and height measurements and blood test for vitamin D. Hypovitaminosis D was defined as serum 25(OH)D < 50 nmol/L. However, 396 (99 %) respondents completed all the requirements for the study.

The questionnaire included age, gender, ethnicity, smoking, educational status, antenatal history, previous medical, surgical, obstetrics and gynecology history, sun protection score, length of time exposed to sunlight (minutes per week) and FFQ vitamin D/My. Sun protection score is the total of sunscreen, scarf (jilbab), caps/hats, long sleeves, gloves, long pants, long skirts and umbrella (minimum = 0, maximum = 8) which are the clothing that women often used outdoors during the day [1]. Meanwhile, length of time exposed to sunlight (minutes per week) equals to multiplying the duration in minutes of sun exposure per day with number of day per week [1]. The other method is to add a vulnerable time exposed to the sun between 10 am to 2 pm within a week. The weight and height of the respondents were measured using a TANITA digital scale and a SECA portable body meter to the nearest 0.1 kg and 0.1 cm, respectively. Two measurements were taken for both weight and height, and the average of the two values were used in the analyses. In this study, we also conducted FSTCM to estimate the type of skin color of the respondents. There are six colors of skin; skin type I (pale white skin), skin type II (white skin), skin type III (light brown skin), skin type IV (moderate brown skin), skin type V (dark brown skin) and skin type VI (black skin) [18]. The FSTCM was placed in the middle of the exposure side of the respondent’s frequently used hand to measure the skin type. This is followed by skin measurement using Mexameter (Mx) 18 *(Courage-Khazaka Electronic GmbH, Germany),* a tool for detecting skin melanin levels easily and quickly. Measurement was performed three times. Researchers were trained to use the device and followed the device protocol. Total serum 25(OH)D was analyzed using electrochemoluminiescent immunoassay (Roche Cobas e411) and followed the standard procedure. In the package insert for cobas e411 (2012), coefficient of variation (CV) of 25(OH)D varied from 2.2–10.7 % [19].

### Statistical analysis

Data were analyzed using the IBM SPSS Statistics Version 22.0. Descriptive values were shown as frequency, mean ± SD or median (interquartile range (IQR 25^th^ and 75^th^)). Simple logistic regression was used in this study to evaluate the association of each independent variable with hypovitaminosis D. *P* value <0.25, clinically or practically important independent variables were included in multivariable logistic regression analysis to obtain the strength of the variables. The prediction model of hypovitaminosis D among pregnant women was reported using adjusted odds ratio, 95% confidence interval and the significant level at *p*-value < 0.05.

## Results

A total of 396 respondents (99 % response rate) aged 18–40 years completed the questionnaire, skin test measurement using FSTCM and Mx 18, anthropometric measurement, and blood test for vitamin D. The mean ± SD age of the respondents was 28.06 ± 4.09 years. A total of 77.5 % were of Malay ethnicity, followed by 11.2 % Chinese, 8.8 % Indian and 2.5 % others (Table 1). A total of 358 (90.4 percent) of respondents had serum 25(OH)D < 50 nmol/L. The median concentration of serum 25(OH) D was 27.11 nmol/L with a minimum value of 7.50 nmol/L and a maximum value of 121.43 nmol/L. Meanwhile, 43.9 percent respondents had serum 25(OH)D < 25 nmol/L. Only five respondents (1.3 %) had enough vitamin D (serum ≥ 75 nmol/L) (Table 2).

**Table 1:** Socio-demographic, antenatal, skin characteristics and vitamin D intake of first trimester pregnant women (*N* = 396)

Independent variables	n	Percent	Mean ± SD / Median (IQR 25 %, 75 %)
A) Socio-demographic factors			
Age (year)			28.06 ± 4.09 (min = 18, max = 40)
Ethnic groups			
Malay	307	77.5	
Chinese	44	11.2	
Indian	35	8.8	
Others	10	2.5	
Educational status			
Primary School	16	4.0	
Secondary School	149	37.6	
Tertiary School	231	58.4	
Working Status			
Working	301	76.0	
Not working	95	24.0	
Household members			3 (2,4) (min = 2, max = 12)
Household income per month			RM3500 (2800,5000) (min = RM900, max = RM20 000)
B) Factors related to antenatal			
Week of pregnancy			10 (8,12) (min = 4, max = 12)
Gravidity			2 (1,3) (min = 1, max = 9)
Parity			0 (0,1) (min = 0, max = 6)
Smoking status			
Yes	3	0.8	
No	393	99.2	
Last Child Birth			
≤ 2 years	124	61.1	
>2 years	79	38.9	
Not applicable (first pregnancy)	193		
Still breastfeeding			
Yes	41	22.9	
No	138	79.1	
Not applicable (no child)	217		
Body Mass Index (BMI) before pregnancy(kg/m^2^)			22.69 (19.95, 26.36) (min = 14.29, max = 53.90)
Weight at first antenatal visit (kg)			57.70 (49.60,68.00) (min = 33.00, max = 148.70)
C) Factors related to skin			
Fitzpatrick skin classification			
Skin type I -II	103	26.0	
Skin type III	173	43.7	
Skin type IV-VI	120	30.3	
Melanin Indices (using Mexameter 18) (0-999.000)			302.17 (245.00, 302.17) (min = 133.33, max = 949.33)
Sun protection score (0-8)			5 (4,5) (min = 0, max = 7)
The length of time exposed to sunlight (minutes per week)			37.50 (0,150) (min = 0, max = 1680)
D) Factors related to vitamin D intake			
Intake of vitamin D in food (IU/day)			202.16 (124.94, 334.62) (min = 13.50, max = 1523.12)
The number of respondents taking antenatal supplements			
Taking supplements containing vitamin D	44	11.1	
Taking supplements without vitamin D intake	6	1.5	
Not taking supplement	346	87.4	
Calcium and vitamin D supplement			
No	386	97.5	
Yes	10	2.5	
Intake of vitamin D from food and supplements (IU/day)			220.04 (137.88, 376.45) (min = 15.10, max = 1523.12)
The level of vitamin D taken from food and supplements (IU/day)			
<200 IU/day	178	44.90	
≥200 IU/day	218	55.10

**Table 2:** Serum vitamin D (25(OH)D) concentrations among first trimester pregnant women (*N* = 396)

Serum vitamin D	n	Percent	Median (IQR 25 %, 75 %)
Serum concentration of 25 (OH)D nmol/L			27.11 (19.87,36.99) (min = 7.50, max = 121.43)
Serum concentration of 25 (OH) D nmol/L (categorized)			
<25.00 nmol/L	174	43.9	
25-49.99 nmol/L	184	46.5	
50-74.99 nmol/L	33	8.3	
≥75 nmol/L	5	1.3	
Serum concentration of 25 (OH) D nmol/L (categorized)			
<50 nmol/L	358	90.4	
≥50 nmol/L	38	9.6	

Table 3 shows the association between each independent variable with hypovitaminosis D. Age, ethnicity, education level, Fitzpatrick classification, melanin indices, sun protection score and intake of vitamin D from food have association with hypovitaminosis D (*p* < 0.05). The crude OR (95%CI) for intake of vitamin D from food was 0.998 (0.997,0.999) with *p* value 0.042. When the data was fixed into two decimal point, it was not significant. The selected independent variables (*p* <0.25) were age, ethnicity, education level, household income per month, household number, weeks of pregnancy, number of children/parity, Fitzpatrick classification, melanin index, sun protection score, vitamin D intake from food (IU/day), vitamin D intake from food and supplement (IU/day), and the level of vitamin D in foods and supplements (IU/day). However, in order to prevent the occurrence of multicollinearity, melanin index, vitamin D intake from food and supplements, and the level of vitamin D in foods and supplements (IU/day) were not included in the multivariable analysis. The Fitzpatrick classification was chosen as the method for skin color measurement because it is simple and easy to measure. Meanwhile, only vitamin D intake from meals was selected as the p value was lower. Employment status, monthly household income, household number, gestational age, parity, last child birth, breastfeeding, pre-pregnancy BMI, weight at first antenatal visit and length of time exposed to sunlight were not significantly associated with hypovitaminosis D (Table 3).

**Table 3:** Combination of descriptive and simple logistic regression analysis to show the relationship between risk factors and hypovitaminosis D (*N* = 396)

Variable		Serum 25(OH)D <50 nmol/L n (%) mean ± SD median (IQR)	Serum 25(OH)D ≥50 nmol/L n (%) mean ± SD median (IQR)	Crude OR (95 % CI)		*p* value
Age(year)^c^		27.91 ± 3.95	29.47 ± 5.07	0.91	(0.84, 0.99)	0.026
Ethnic groups^c^	Malay	296 (96.40)	11 (3.60)	22.42	(9.63, 52.21)	<0.001
	Chinese	24 (54.50)	20 (45.5)	1.00		
	Indian	32 (91.40)	3 (8.60)	8.89	(2.36, 33.40)	0.001
	Others	6 (60.00)	4 (40.00)	1.25	(0.31, 5.06)	0.754
Education level^c^	Primary	11 (68.80)	5 (31.20)	1.00		
	Secondary	136 (91.30)	13 (8.70)	4.75	(1.43, 15.80)	0.011
	Tertiary	211 (91.30)	20 (8.70)	4.80	(1.52, 15.18)	0.008
Working Status	Working	275 (91.40)	26 (8.60)	1.00		0.252
	Not working	83 (87.40)	12 (12.60)	0.65	(0.32, 1.35)	
Household income per month^c^		3500 (2800,5000)	3750 (2500,5250)	1.00	(1.00, 1.00)	0.060
Household members^c^		3 (2,4)	3 (2,5)	0.90	(0.75, 1.07)	0.225
Weeks of pregnancy at first antenatal visit^c^		10.0 (8.0,12.0)	11.5 (8.0,12.0)	0.89	(0.77,1.02)	0.100
Parity^c^		0 (0,1)	1 (0,1)	0.79	(0.61, 1.03)	0.083
Last child birth	≤ 2 years	111 (89.50)	13 (10.50)	0.86	(0.43,1.75)	0.686
	>2 years^a^	247 (90.80)	25 (9.20)	1.00		
Breastfeeding	Yes	36 (87.80)	5 (12.20)	0.74	(0.27,2.01)	0.552
	No^b^	322 (90.70)	33 (9.30)	1.00		
BMI before pregnant (kg/m^2^)		22.76 (20.00,26.25)	22.15 (19.75,26.58)	1.02	(0.96,1.09)	0.499
Weight at first antenatal visit (kg)		57.85 (49.30,68.54)	57.03 (51.49,65.61)	1.01	(0.98,1.03)	0.569
Fitzpatrick Classification^c^	Skin type I-II	83 (80.60)	20 (19.40)	1.00		
	Skin type III	163 (94.20)	10 (5.80)	3.93	(1.76,8.77)	0.001
	Skin type IV-VI	112 (93.30)	8 (6.70)	3.37	(1.42,8.03)	0.006
Melanin Indices (using Mexameter)	(0-999.000)	306.17 (254.17,373.67)	242.83 (181.08, 354.00)	1.01	(1.00,1.01)	0.006
Sun protection score	(0-8)^c^	5 (4,5)	3.5 (1,5)	1.77	(1.42,2.21)	<0.001
Total of respondents exposed to sunlight (10 am-2 pm)	Yes	201 (90.10)	22 (9.90)	1.00		
	No	157 (90.80)	16 (9.20)	1.07	(0.55,2.11)	0.836
The length of time exposed to sunlight (minutes in week)		35 (0,150)	55 (0,210)	1.00	(1.00,1.00)	0.570
Intake of vitamin D from food (IU/day)^c^		197.51 (122.70,332.05)	263.81 (142.90,460.55)	1.00	(1.00,1.00)	0.042
Intake of vitamin D from food and supplements (IU/day)		215.080 (132.93,361.98)	302.135 (147.89,515.74)	1.00	(1.00,1.00)	0.064
The level of vitamin D in foods and supplements (IU/day)						
<200 IU/day		165 (92.70)	13 (7.30)	1.64	(0.82,3.32)	0.165
≥200 IU/day		193 (88.50)	25 (11.50)	1.00		

*OR* odds ratio, *CI* Confidence interval

*p* values have been derived from Wald tests

^a^Respondents with last child birth more than 2 years and respondents who did not have previous child (primid)

^b^Respondents who did not breastfeed during pregnancy and those who did not have children.

^c^Independent variables included in the multiple logistic regression

1.00 as a reference group

During the multivariable analysis there was no multicollinearity or interaction between variables. Hosmer Lemeshow goodness-of-fit was not significant and no outliers. Receiver Operating Characteristics (ROC) area under the arches of this study was 0.866 with p <0.001 and can accurately predict 86 % cases. The final model is shown in Table 4. Interpretation of the final model is as follows; 1) Malay pregnant women in first trimester have a 34 times higher risk to have hypovitaminosis D compared to Chinese pregnant women, 2) Indian pregnant women in the first trimester have a 17 times higher risk of hypovitaminosis D compared to Chinese pregnant women, and 3) Pregnant women in the first trimester with a secondary or tertiary level of education have 12 times and 14 times higher risk respectively to have hypovitaminosis D compared to pregnant women with a primary level of education. The forecast model of having hypovitaminosis D among pregnant women in the first trimester was: logit (P) = ln [P/1-P] = -1.61 + [3.52*Malays] + [2.82* Indians] + [2.50*secondary school education] + [2.67*tertiary school education] (Table 4).

**Table 4:** Multiple logistic regression analysis to determine the predictors of hypovitaminosis D (serum 25(OH) D < 50 nmol/L)

Independent variable	B	Adjusted OR	(95 % CI)	*p* value
Ethnic groups				
Chinese		1.00		
Malay	3.52	33.68	(2.81,88.56)	<0.001
Indian	2.82	16.86	(3.78,75.20)	<0.001
Education level				
Primary		1.00		
Secondary	2.50	12.12	(2.71, 54.16)	0.001
Tertiary	2.67	14.38	(3.31, 62.45)	<0.001
Constant	−1.608	0.200		0.032

B- Regression coefficient, *OR* odds ratio, *CI* confidence Interval, *df* degree of freedom

1.00 as a reference group

## Discussion

In this study, the prevalence of hypovitaminosis D among pregnant women with various ethnicities and skin colors was high. Hypovitaminosis D is expected to be high as previous studies related to vitamin D conducted in Malaysia among children and Malay women were also high. The prevalence of hypovitaminosis D (serum 25 (OH) D < 50 nmol/L) among children aged 7 to 12 years old in primary schools in Kuala Lumpur was 70.4 % [3]. Whereas, for Malay women (222 respondents) with mean ± SD age 47.7 ± 4.6 years, their mean ± SD serum 25(OH) D was 36.2 ± 13.4 nmol/L [19]. This high prevalence of hypovitaminosis D was due to age, abdominal obesity and metabolic syndrome affecting vitamin D levels. The mean ± SD serum 25(OH) D among Malay ethnic in our study was 27.4 ± 12.5 nmol/L with mean ± SD age 27.6 ± 3.9 years. The serum vitamin D level was low probably because our respondents were first trimester pregnant women who may have morning sickness and limited outdoor physical activity. Hamid et al. [20] reported that the prevalence of hypovitaminosis D (25 (OH) D serum <50 nmol/L) among pregnant women in the second and third trimester was 60 and 37 % respectively. The prevalence declined due to a significant increase in the intake of multivitamins in the third trimester [20]. This is in line with our study, where the prevalence of hypovitaminosis D (25 (OH) D serum <50 nmol/L) in the first trimester was high (90.4 %) and only 11.1 % took supplements containing vitamin D. Moreover, most of them had just started the supplements and the results may not have affected their vitamin D status yet.

Maghbooli et al. [21] reported that the prevalence of vitamin D deficiency (serum 25(OH)D <25 nmol/L) among pregnant women in Tehran was 70.6 % and the prevalence of severe vitamin D deficiency (serum 25(OH)D <12.5 nmol/L) was 28.8 %. They classified the level of serum 25 (OH) D into four groups; below 12.5 nmol/L as severe, 12.5 to 24.9 nmol/L as moderate, 25 to 34.9 nmol/L as mild, and more than 34.9 nmol/L as normal [21]. In comparison, our results showed a much lower prevalence of vitamin D deficiency with 43.9 % having serum 25(OH)D <25 nmol/L (Table 2). This may be due to the multiethnic nature of our respondents. Ethnic Chinese in this study had better vitamin D results compared to Malays and Indians which contribute to the decrease of the overall measure. The low vitamin D status in Tehran was probably due to wearing of full dress and veil among all women, regardless of race and religion.

Data related to the prevalence of hypovitaminosis D among pregnant women in Southeast Asia is limited. Hien et al. [10] reported that among pregnant women and those of reproductive age (15–49 years old) in Vietnam, the prevalence of having serum 25(OH)D < 50 nmol/L and < 75 nmol/L was 7 and 48 % respectively. The prevalence of hypovitaminosis D (serum 25(OH)D < 50 nmol/L) was low because the respondents comprised of both pregnant and non pregnant women, from a mix of urban and rural district, with some working in the farming sector. These contrasted with our study where our samples were pregnant women who lived in an urban district, in which none of them were farmers.

Charatcharoenwitthaya et al. [22] stated that among pregnant women in Thailand, serum 25(OH)D < 75 nmol/L in the first trimester was 83.3 %, 30.9 % in the second trimester and 27.4 % in the third trimester. There were no predictive factors for serum 25(OH)D < 75 nmol/L in the first trimester. In the second trimester, there were two factors that predict inadequate vitamin D, namely not taking antenatal vitamins (OR 4.28; 95 % CI: 1.33–13.75; *p* < 0.05) and inadequate serum vitamin D levels in the first trimester (OR 5.10; 95 % CI: 1.93–13.47; *p* < 0.01). Predictors of low vitamin D among the third trimester pregnant women were they did not take milk that has been fortified with vitamin D (OR 11:42; 95 % CI: 3.12–41.86), did not take antenatal vitamin supplements (OR 9.70; 95 % CI: 2.28–41.19) and had low vitamin D level in the first trimester (OR 10.58; 95 % CI: 2.89–38.80). Lack of vitamin D was not found in pregnant women who consumed antenatal vitamin supplements. However, 20 pregnant women who consumed antenatal vitamins with vitamin D at least 400 IU/day still had inadequate vitamin D levels in the third trimester [22]. Charatcharoenwitthaya et al. [22] suggested that vitamin D deficiency is common among pregnant women in Thailand, especially in the first trimester [16]. Their study was similar to ours, where intake of vitamin D supplement or prenatal vitamins containing vitamin D in the first trimester was not associated with adequate serum 25(OH)D level. This was due to only 11.1 % respondents took vitamin D supplements or antenatal vitamin supplements containing vitamin D in the first trimester.

Pérez-López et al. [23] stated that the prevalence of hypovitaminosis D (serum 25(OH)D <50 nmol/L) for pregnant women in Spain was 22.7 %. The study revealed that the factors associated with hypovitaminosis D were season of sampling, nulliparity, maternal weight and non-Caucasian ethnic groups [23]. The prevalence of hypovitaminosis D (serum 25 (OH) D <50 nmol/L) in our study was four times higher than Spain. The difference may be due to factors such as sample size, methodology, study design, sampling season, as well as ethnic and genetic characteristics of the participants, and/or other factors which may affect the biological diversity of the results.

Our main problem is the variation in the definition of vitamin D deficiency. The laboratory measurement techniques for measuring vitamin D are also different. In order to standardize the level of vitamin D, cut off point for serum 25(OH) D of <50 nmol/L was used for comparison between various studies. This concentration has been used in other vitamin D studies in Malaysia [1, 2, 24]. In fact, there are a few studies abroad that also used the term hypovitaminosis D as serum 25(OH)D <50 nmol/L [25–27].

Chailurkit et al. [28] stated that hypovitaminosis D was higher among adult in Thailand. Their research involved a Thai population aged 15 to 98 years with a sample size of 21 960. Their low serum 25(OH)D was associated with young urban women. Meanwhile, the mean ± SD serum 25(OH)D in their study was 73.0 ± 0.8 nmol/L [28], which is far greater than the median (IQR) serum 25(OH)D in our study which is 27.11 (19.87, 36.99) nmol/L. This may be related to predictors used by Chailurkit et al. [28] that were included in our study selection criteria. Our study was conducted in an urban district in the first trimester of pregnancy among mothers aged between 18 and 40 years. The significant predictive factors after multivariable analysis in our study were ethnic group, educational status and dietary intake of vitamin D. However, the study by Chailurkit et al. [28] covered both urban and rural areas.

The majority of respondents in our study were Malay pregnant women (77.5 %) aged around 28 years old with a high level of education (58.4 %), working (76.0 %), with a household number of three and a total family income around RM3500. Ethnicity plays an important role and was one of the predictors for hypovitaminosis D in our study. Green et al. [2] found that Chinese women in Kuala Lumpur had 14 nmol/L higher concentrations of serum 25 (OH) D with a lower prevalence of hypovitaminosis D compared to Indians and Malays. Rahman et al. [29] also mentioned similar findings, where the level of serum 25(OH)D was significantly lower in postmenopausal Malay women (44.4 ± 10.6 nmol/L) compared to Chinese women (68.8 ± 15.7 nmol/L). In our study, first-trimester Malay and Indian pregnant women had 34 times and 17 times higher probability of hypovitaminosis D respectively compared to Chinese pregnant women. This finding may be related to sun protection factor and skin color. Majority of Malay respondents have skin color type III, Indian respondents have skin color type IV and Chinese respondents have skin color type II. Mean ± SD sun protection for Malay, Indian and Chinese respondents were 4.73 ± 0.76, 3.17 ± 1.20 and 2.02 ± 1.41 respectively (not in table). After the analysis of the data among Malay ethnicity, the risk factors for hypovitaminosis D were health education status (secondary education) and high sun protection score.

In term of education, 4 % of pregnant women only attended primary school education. This result was expected as the samples were recruited from urban areas. Leffelaar et al. [30] claimed that in their study, among 4236 pregnant women in Amsterdam, there was a significant association between education level and serum 25 (OD) levels. They reported that women with low education have lower serum 25(OH)D compared to highly educated women (*p* < 0.001). This result is also similar to the study by Van Den Berg et al. [31], where in their cohort study of 2274 pregnant women in the Netherlands, serum 25(OH)D level in early pregnancy was significantly and positively associated with education level. Logistic regression analysis was carried out to show that women with lower education had lower serum 25(OH) D and higher risk of small for gestational age (SGA) fetuses (OR 1.95 [95 % CI; 1.20–3.14]). Holvik et al. [32] also claimed that increased duration of education was associated with increased serum 25(OH)D among women. The association was not valid for men [32]. Rock [33] reported that a higher level of education, physical activity and a lower BMI were associated a higher intake of multivitamins-multiminerals.

Educational status was one of the significant predictors for hypovitaminosis D in our research, albeit with a negative association. Our findings showed that primary education predicted a higher serum 25(OH)D, in contradiction with previous studies. This is likely due to more exposure of respondents with lower education to sunlight in their daily activities as a result of poorer amenities such as lack of protective private transportation (e.g car) and holding a manual job with prolonged solar exposure. Sunscreen cream may be a luxury not available to this group of women during outdoor activities. However, this assumption would not be effective for countries with less exposure to the sun, where low education was associated with lower serum 25(OH)D, perhaps as a result of less dietary intake of vitamin D and less opportunity for sunbathing leisure and vacation. Hien et al. [10] claimed that work status (farmer versus non farmer) have significant impact on vitamin D levels, where farmers had higher levels of serum 25(OH)D compared to non-farmers. However, their study did not mention the association between educational level and serum 25(OH) D.

Mithal et al. [26] concluded from other studies in developing countries that economic status and high family income showed a lower prevalence of hypovitaminosis D in all age groups. High-income families provided better outdoor activities for school teenagers, better antenatal care facilities for pregnant women, consumed balanced diet and could afford vitamin D supplements for their older people. Most of the studies claimed that there was no association between household income per month and vitamin D status [34]. This was similar to our study where household income was not associated with hypovitaminosis D. Our respondents were first trimester pregnant women, some of them were having morning sickness that causes poor dietary intake. They probably rarely spent their time outside home and avoided exposure to the sun. Their unstable condition prevented them from having a balanced diet and being active despite a high household income.

Our study reported that 51.3 % of respondents were pregnant more than once, 54.8 % had no children, 99.2 % did not smoke, 61.1 % had last child birth two years ago and below, median (IQR) BMI before pregnancy was 22.69 kg/m^2^ (19.95, 26.36) and median (IQR) body weight in the first antenatal visit was 57.70 kg (49.60, 68.00). None of the antenatal history associated with vitamin D status. Charatcharoenwitthaya et al. [22] also claimed that there was no significant relationship between pre-pregnancy BMI parity or gravidity with insufficient vitamin D level in any trimester. Clifton-Bligh et al. [25] who conducted a study on 307 pregnant women in Australia also showed similar results where the weight (*p* = 0.87) or BMI (*p* = 0.35) was not associated with serum 25(OH)D levels.

However, several studies showed associations between BMI and serum 25 (OH) D. The higher the BMI, the lower the serum 25(OH)D [1, 3, 13]. This was not found in our study as most of our respondents have normal weight, aged about 28 years and were in their first pregnancy. Wortsman et al. [35] reported that BMI had a negative moderate correlation with serum 25(OH)D after irradiation (*r* = -0.55, *p* = 0.003). They also claimed that obesity was related with hypovitaminosis D [35]. This may be due to a decreased ratio of body surface area to bulk with increased body weight and deposition of nutritional elements such as vitamin D in body fat.

Majority of our respondents had skin type III, median (IQR) melanin indices of 302.17 (245.00, 302.17), median sun protection score of 5 (4,5) and length of time exposed to sunlight (minutes per week) of 37.50 (0150) minutes. We have divided skin type into three classes, skin types I-II, skin type III and skin types IV-VI. This classification is based on the Fitzpatrick skin reactions when exposed to the sun. Skin type I-II is pale or fair, flammable and rarely turns dark/tan. Skin type III is flammable and turns dark, and is the commonest type in the United States. Skin types IV to VI, do not burn but easily turns dark [18]. Many previous studies reported that skin color type was associated with serum 25(OH)D. Clemens et al. [36] claimed that increased skin pigmentation can reduce the transmission of ultraviolet rays to synthesize vitamin D3 and this leads to a high risk of vitamin D deficiency. Armas et al. [37] also stated the same opinion that any effect of ultraviolet (UV) on the level of serum 25 (OH) D depends on skin pigmentation and the amount of UV received. Our study showed similar results where fair skin such as Chinese had a higher level of serum 25(OH) D. Initially, by using simple logistic regression analysis, the skin color type, melanin index measurement and sun protection score showed correlation with vitamin D status (*p* < 0.05). After using multivariable analysis (binary logistic regression), the final model showed that ethnicity is a predictor of serum 25(OH) D levels. Malay ethnicity was associated with high sun protection score, while Indian ethnicity was associated with high skin pigmentation. Clifton-Bligh et al. [25] also reported that serum 25(OH) D was associated with ethnicity, whereby serum 25(OH) D levels were lower in women who originated from the Indian subcontinent and the Middle East.

Self-reported duration of time exposed to sunlight (minutes per week) in this study showed that respondents tend to spend more time indoors than outdoors between 10 am until 2 pm in the afternoon. Of 396 respondents, 223 (56.31 percent) were exposed to sunlight between 10 am and 2 pm. Although more than half of respondents were exposed to sunlight, most of them were exposed for only a short period of time with median (IQR) length of time exposed of 37.50 (0150) minutes in a week. In addition, most Malaysians avoid going out at that particular time of day because of the hot and sunny weather. Certain dress code and sun block had limited the ability of the skin to synthesize vitamin D.

Vitamin D intake from food is one of the modifiable factors that can reduce the risk of hypovitaminosis D. A total of 55.1 % of respondents have met the Malaysian Recommended Nutrient Intakes (RNI) for vitamin D in pregnant women which require intake of 5 μg (200 IU) per day [38, 39] (Table 1). Although over half of respondents had met the RNI for vitamin D (≥200 IU/day), many respondents still had serum 25(OH)D < 50 nmol/L. The RNI is considered too low as many pregnant women had serum 25(OH)D < 50 nmol/L. Meanwhile, the Institute of Medicine [40] reported that the Recommended Dietary Allowance (RDA) of vitamin D for pregnant mothers is 600 IU/day. Charatcharoenwitthaya et al. [22] also claimed that there was a significant difference in serum 25(OH)D between pregnant women who took 400 IU or more vitamin D as supplements during the antenatal period and pregnant women who took < 400 IU/day or none (OR 0.19; 95 % CI: 0.06 to 0.56, p <0.01).

There are several limitations in this study, one of which was recall bias, as data was obtained through interview. In order to avoid such problems, a trained interviewer would often use different questions with the same meaning or repeat the questions several times. The other issue was the compliance to the antenatal vitamins prescription as there were no pill calculations. The confidence interval was relatively huge for ethnic groups. Although the overall sample size was sufficient, the sample size for Chinese and Indian were not adequate. Malaysian survey carried out in 2010 claimed that the Malays have high fertility rate of 2.8 children per woman, compared to 1.8 and 2.0 children among every Chinese and Indian woman respectively. The prevalence of hypovitaminosis D (serum < 50 nmol/L) may not be comparable to other populations as our respondents were first trimester pregnant women staying in town with no involvement in any agricultural activity.

Finally, awareness of high prevalence and dangers of hypovitaminosis D during pregnancy needs to be highlighted to health care workers so that they are better informed of factors influencing vitamin D status. Health care workers should also know the functions and the sources of vitamin D, so that advice could be given to patients especially those with lactose intolerance or low milk consumption during pregnancy. Calcium supplement with vitamin D should be given earlier to those who do not consume adequate milk during pregnancy to prevent adverse health effects to the mother and fetus.

## Conclusion

The study revealed high prevalence of hypovitaminosis D among Malaysian urban pregnant women in the first trimester. Dietary intake of food containing vitamin D was found to reduce hypovitaminosis D. Nutrition education needs to be emphasized among pregnant women, especially those of Malay and Indian ethnicity, who consume little or no milk, and those who work at desk jobs as indicated by high education levels. Further studies need to be conducted among health care staff to assess the level of knowledge related to vitamin D. In addition, this research aimed to create the awareness on the importance of vitamin D to patient.

## Abbreviations

FFQ vitamin D/My, validated semi-quantitative food frequency questionnaire for vitamin D in Malaysia; FSTCM, fitzpatrick skin type chart measurement; IQR, interquartile range; MI, melanin indices; Mx, mexameter; SD, standard deviation
